# Modelling the evolution of drug resistance in the presence of antiviral drugs

**DOI:** 10.1186/1471-2458-7-300

**Published:** 2007-10-23

**Authors:** Jianhong Wu, Ping Yan, Chris Archibald

**Affiliations:** 1 Center for Disease Modeling, Department of Mathematics and Statistics, York University, 4700 Keele Street, Toronto, M3J 1P3, Canada; 2 Surveillance and Risk Assessment Division, Centre for Infectious Diseases Prevention and Control, Public Health Agency of Canada, AL: 0602B – Tunney's Pasture, Room: 2351 – 100 Eglantine Driveway, Ottawa, K1A 0K9, Canada

## Abstract

**Background:**

The emergence of drug resistance in treated populations and the transmission of drug resistant strains to newly infected individuals are important public health concerns in the prevention and control of infectious diseases such as HIV and influenza. Mathematical modelling may help guide the design of treatment programs and also may help us better understand the potential benefits and limitations of prevention strategies.

**Methods:**

To explore further the potential synergies between modelling of drug resistance in HIV and in pandemic influenza, the Public Health Agency of Canada and the Mathematics for Information Technology and Complex Systems brought together selected scientists and public health experts for a workshop in Ottawa in January 2007, to discuss the emergence and transmission of HIV antiviral drug resistance, to report on progress in the use of mathematical models to study the emergence and spread of drug resistant influenza viral strains, and to recommend future research priorities.

**Results:**

General lectures and round-table discussions were organized around the issues on HIV drug resistance at the population level, HIV drug resistance in Western Canada, HIV drug resistance at the host level (with focus on optimal treatment strategies), and drug resistance for pandemic influenza planning.

**Conclusion:**

Some of the issues related to drug resistance in HIV and pandemic influenza can possibly be addressed using existing mathematical models, with a special focus on linking the existing models to the data obtained through the Canadian HIV Strain and DR Surveillance Program. Preliminary statistical analysis of these data carried out at PHAC, together with the general model framework developed by Dr. Blower and her collaborators, should provide further insights into the mechanisms behind the observed trends and thus could help with the prediction and analysis of future trends in the aforementioned items. Remarkable similarity between dynamic, compartmental models for the evolution of wild and drug resistance strains of both HIV and pandemic influenza may provide sufficient common ground to create synergies between modellers working in these two areas. One of the key contributions of mathematical modeling to the control of infectious diseases is the quantification and design of optimal strategies, combining techniques of operations research with dynamic modeling would enhance the contribution of mathematical modeling to the prevention and control of infectious diseases.

## Background

The emergence of drug resistance in treated populations and the transmission of drug resistant strains to newly infected individuals are important public health concerns in the prevention and control of infectious diseases such as HIV and influenza. Mathematical modelling may help guide the design of HIV treatment programs to minimize the development and spread of drug resistant strains. Modelling may also help us achieve a better understanding of the optimal use of antiviral drugs as the first line of defence against a new strain of influenza and of the potential benefits and limitations of mitigation strategies using antiviral drugs during an influenza pandemic.

While acknowledging the fundamental differences in the evolution of drug resistance between the two diseases, sufficient common ground may be found to create synergies between modellers working in these two areas. To explore this further, the Public Health Agency of Canada (PHAC) and Mathematics for Information Technology and Complex Systems (MITACS, one of the centres of the Network of Centres of Excellence funded by the Government of Canada) organized a workshop in Ottawa in January 2007. The workshop brought together selected scientists and public health experts to discuss the emergence and transmission of HIV antiviral drug resistance, to report on progress in the use of mathematical models to study the emergence and spread of drug resistant influenza viral strains, and to recommend future research priorities.

In the opening remarks, **Dr. Chris Archibald (**Surveillance and Risk Assessment Division, PHAC) indicated that PHAC strongly supports collaboration between modelers and public health policy makers to better understand the evolution of drug resistant strains and that the research priority recommendations coming out of the meeting would be closely examined.

### HIV Drug Resistance at the Population Level

**Dr. Donald Sutherland (**HIV Drug Resistance Strategy, WHO) spoke about the work of WHO in the area of HIV drug resistance (DR) and the approach to HIVDR within the global goal of universal access to anti-retroviral treatment by 2010. He stressed the importance of addressing the potential problem of drug resistance during the scale-up of anti-retroviral treatment programs. WHO's program of HIVDR monitoring follows individuals who are starting HIV treatment for the first time and makes use of a variety of early warning indicators that may signal a potential problem with drug supply or treatment compliance, and hence a greater likelihood of the development of HIV drug resistance. HIVDR transmission surveillance is done through a threshold survey of a small number of recently infected persons to assess transmitted HIV drug resistance; the percentage of persons that show laboratory evidence of DR is used to decide whether to trigger a set of specific recommended actions (with 5% being the first threshold for considering action). Dr. Sutherland concluded by providing a number of useful WHO web links for HIV treatment and drug resistance information [1-6].

In a lecture entitled "Predicting the Unpredictable: the Evolution of Drug Resistant HIV Epidemic in Africa", **Dr. Sally Blower **(Disease Modeling Group, Semel Institute for Neuroscience and Human Behavior, UCLA) echoed the concern that the scale-up of anti-retroviral treatment could generate an epidemic of drug resistant HIV. She reviewed her previous modelling work on HIV and anti-retroviral treatment [7-9] that assesses the dynamics of wild-type and DR-strains in a population for which a portion of infected individuals receive treatment. For developed countries where treatment rates are high, her model-based analysis showed that although treatment increased the transmission of DR, it significantly decreased HIV transmission and prevalence and thus could eventually lead to eradication of the HIV epidemic [7,10,11]. However, for developing countries where treatment rates are low (such as for many countries in Africa), there is little effect on HIVDR transmission and HIV incidence rates are unlikely to change. WHO is designing a HIVDR transmission surveillance system in Africa to detect whether transmitted resistance will exceed the threshold of 5%, but Dr. Blower's analysis for Botswana showed that this threshold may not be reached for a long time [12-15].

Dr. Sally Blower's lecture also illustrated how mathematical modelling can be used to help determine the optimal use of limited resources. She presented recent work [16] that used operations research techniques to determine the optimal strategy for allocating anti-retroviral drugs among health care facilities to ensure that each individual infected with HIV has an equal chance of receiving treatment.

### HIV Drug Resistance in Western Canada

**Dr. Gayatri Jayaraman **(HIV Drug Resistance and Field Surveillance Section, PHAC) introduced the Canadian HIV Strain and DR Surveillance Program. She described the methods used to collect samples from individuals newly diagnosed with HIV and to analyze *pol *gene sequences to determine drug resistance mutations according to the International AIDS Society-USA list (2005). She stressed the uniqueness of this surveillance program since it gathers data at the population level rather than from a limited number of clinical sites. **Dr. Ping Yan **(Modelling & Projection Section, PHAC) then presented his exploratory analysis of trends in transmitted drug resistance in western Canada, and **Dr. Shenghai Zhang **(Modelling & Projection Section, PHAC) discussed further stratification of the population and its impact on the trend analysis.

In summary, this work modeled trends in the prevalence of transmitted drug resistance using data collected from this surveillance program for the four provinces of western Canada between 1998 and 2004. Generalized linear models were fit to the binary data to examine trends in drug resistance for three drug classes: nucleoside reverse transcriptase inhibitors (NRTIs), non-nucleoside reverse transcriptase inhibitors (NNRTIs), and protease inhibitors (PIs). The results showed that while the overall prevalence of transmitted drug resistance has remained constant over time, there were significant differences according to drug class: a decrease in the prevalence of transmitted NRTI resistance, an increase in the prevalence of transmitted NNRTI resistance, and little change in the prevalence of transmitted PI resistance. In addition, there were distinct differences between provinces in these trends. Possible reasons for these differences over time and between provinces include changes in drug prescribing patterns and in risk behaviours. In conclusion, the results highlight the need for continued national surveillance of transmitted drug resistance to fully understand inter-regional differences and the course of the HIV epidemic in Canada [17].

### HIV Drug Resistance at the Host Level: Optimal Treatment Strategies

Four lectures dealt with HIV drug resistance and optimal treatment strategies.

**Dr. John Mittler **(Department of Microbiology, University of Washington) described two kinds of regimen-sparing treatment strategies: structured treatment interruptions and induction-maintenance therapies. He then presented his model-based analysis to help determine the optimal length for the induction phase, ways to improve the success of induction-maintenance therapy, and which drugs should be included in the induction and maintenance regimens [18,19].

**Dr. Robert Smith? **(Departments of Mathematics and of Epidemiology, University of Ottawa) emphasized that mathematical models for HIV treatment should explicitly account for the mechanics and temporal aspects of drug dosing. His analysis suggested that the use of PIs alone may lead to treatment failure, whereas RTIs would lead to treatment success, whether or not they were combined with PIs. He also presented his recent work which shows that to minimize drug resistance, RTI therapy should remain outside a certain (predictable) parameter region [20,21].

In his lecture entitled "Emergence and Impact of HIV CTL-Escape Mutants on Progression to AIDS", **Dr. Beni Sahai (**Cadham Provincial Laboratory, Manitoba) presented several theories for the loss of effectiveness of the anti-HIV immune response, and concluded that anti-HIV cytotoxic T-lymphocytes (CTLs) play an essential role in preventing progression to AIDS and that drug-resistant HIV strains do not cause a surge of viremia or accelerate progression to AIDS in the presence of multivalent anti-HIV CTLs.

**Dr. Jane Heffernan **(Department of Mathematics and Statistics, York University) noted that viral load and CD4 T-cell counts in patients infected with HIV are commonly used to guide clinical decisions regarding drug therapy or to assess therapeutic outcomes in clinical trials. However, random fluctuations in CD4 T-cell count and viral load, due solely to the stochastic nature of HIV infection, can obscure clinically significant change. She reported her work [22-24] that employed a Monte Carlo simulation to investigate the contributing factors in the expected variability in CD4 T-cell count and viral load. It was found that, by considering correlations between viral load measurements (in the absence or presence of drug therapy) taken days or weeks apart, the variability predicted by the Monte Carlo simulation may reconcile the wide range of variability in viral load observed in clinical studies. Dr. Heffernan discussed how the Monte Carlo simulation can be extended to investigate the effects of drug resistance, and noted that the extended model can estimate the probability that a drug resistant mutant will emerge before and during therapy, the speed at which the drug resistant mutant will dominate once it has emerged, and the probability that drug therapy will clear the wild type and mutant viral strains in a small volume of plasma. The simulation model can also be used to investigate the effects of imperfect treatment adherence on the emergence of drug resistance and to quantify the role of the latently-infected cell pool in harboring drug resistant virus.

### Drug Resistance for Pandemic Influenza Planning

Influenza pandemics have historically been devastating to human populations. Considering the extent of morbidity and mortality caused by previous pandemics and the growing threat of an imminent human outbreak of the avian influenza strain H5N1, identification of effective mitigation strategies is a major global public health priority. It has been recognized that pharmaceutical measures (vaccines, antiviral drugs) would have the greatest impact in containing a pandemic. However, given the significant challenges involved in the development of an effective vaccine against a newly emerging pandemic strain, it is likely that antiviral drugs will be the sole pharmaceutical defense. Since a critical limitation to their application is the emergence of highly transmissible resistant viral mutants, it is paramount to evaluate the use of these drugs for both treatment and prophylaxis. To do this, mathematical models need to be developed that not only incorporate the population phenomenon of disease transmission, but also integrate viral evolution and disease dynamics at the individual level.

**Dr. Seyed Moghadas **(Institute for Biodiagnostics, National Research Council of Canada) described a model to evaluate the potential impact of an antiviral drug strategy on the emergence of drug-resistance and containment of an influenza pandemic [25]. The model-based simulations reveal that elimination of the wild-type strain depends on the timely application of drugs for treatment of index cases and on the level of population coverage by treatment, and suggest that a single strategy of antiviral treatment will be successful only if the reproductive number of the wild-type strain is below 1.9. The results also showed that the early application of antiviral drugs is crucial to contain a pandemic, and caution is required to prevent the emergence of drug-resistant viral mutants since the evolution of host-pathogen systems occurs on a short time scale. Resistant strains with sufficiently small reproductive numbers are soon out-competed, and effective treatment may therefore result in disease elimination. However, it is possible that a resistant strain undergoes further mutations that result in increased transmission fitness. Examination of the past three pandemics suggests that an outbreak of resistant virus is likely to occur if the relative transmission fitness of the resistant virus (relative to wild type) is above 0.4. The results also indicate that for wild-type viruses with high reproductive numbers, the control of a pandemic with antiviral treatment requires a sufficiently low transmission fitness of resistant virus to rule out the occurrence of an outbreak of resistant virus. The issue of DR in relation to the use of antivirals for prophylaxis was also discussed briefly, but this topic clearly requires further study.

**Dr. Murray Alexander **(Institute for Biodiagnostics, National Research Council of Canada) compared deterministic models with individual-based network models [26-29] and stressed the importance of developing something between these two extremes due to the uncertainty of parameter identification and the dependence of current pandemic models on social network structure.

**Dr. John Glasser **(Centers for Disease Control and Prevention, USA) spoke about the evaluation of alternative vaccination and treatment strategies. He presented a standard compartmental model in which infection rates reflected different social activities and networking by age-group [30]. The impact on mortality among the elderly of vaccinating school children exceeds that of vaccinating the older adults themselves, and the impact on mortality among infants was even greater. He noted that in a pandemic, anti-viral medications would be scarce and targeting their use could help ensure their efficient use and also mitigate the evolution of drug resistance.

In a discussion facilitated by **Drs. Fred Brauer **(Department of Mathematics, University of British Columbia) and **Zhilan Feng **(department of Mathematics, Purdue University), **Dr. Theresa Tam **(Immunization and Respiratory Infections Division, PHAC) suggested that pandemic influenza plans should incorporate other non-medical measures (such as social distancing) and she encouraged further work that examines the drug resistance issue for scenarios involving prophylaxis as well as treatment.

### Round-table Discussions: Next Steps

The workshop ended with a group discussion on next steps coordinated by **Dr. Jianhong Wu **(Department of Mathematics and Statistics and Center for Disease Modeling, York University and MITACS). He first invited Dr. Jayaraman to comment on the HIV issues that should be addressed through modeling and Dr. Jayaraman listed some of the areas where she and her colleagues at PHAC thought input from modelers would be most useful:

1. To predict the incidence and prevalence of transmitted (and acquired) drug resistance;

2. To model the contribution of transmitted DR to overall DR HIV prevalence;

3. To model the contribution of transmitted DR to annual HIV incidence;

4. To model which factors contribute the most to transmitted (and acquired) DR and what factors that can minimize the transmission of DR;

*5. To model the level of increased risk behaviour that would off-set the expected decrease in HIV incidence resulting from widespread anti-retroviral therapy use*.

The general discussion suggested that some of these issues can possibly be addressed using existing mathematical models, with a special focus on linking the existing models to the data obtained through the Canadian HIV Strain and DR Surveillance Program. Participants noted that the preliminary statistical analysis of these data led by Dr. Yan has already provided some solutions to items #1–4 in terms of historical data. This, together with the general model framework discussed in Dr. Blower's lecture, should provide further insights into the mechanisms behind the observed trends and thus could help with the prediction and analysis of future trends in the aforementioned items. Participants also noted the remarkable similarity between dynamic, compartmental models for the evolution of wild and drug resistance strains of both HIV and pandemic influenza, and so the above remarks also apply to the drug resistance issue for pandemic influenza. It was suggested that proposals should be prepared to apply for funding to develop new models and take advantage of the large amount of high quality surveillance data available at PHAC.

The participants felt the urgent need of high quality clinical data for the study of drug resistance and it was believed that future collaboration between modelers and public health policy makers would benefit very much from a centralized clinical data base. Therefore, it was recommended that such a data base should be developed as soon as possible.

Finally, participants observed that one of the key contributions of mathematical modeling to the control of infectious diseases is the quantification and design of optimal strategies. This was demonstrated in all the lectures of the workshop and the proposed priority item #5 further reinforced this observation. Combining techniques of operations research with dynamic modeling would enhance the contribution of mathematical modeling to the prevention and control of infectious diseases.

## Competing interests

Mathematics for Information Technology and Complex Systems (MITACS) provides the funding support to cover the article processing cost. Otherwise, the authors declare that they have no competing interests.

## Authors' contributions

JW, PY and CA helped conceive the workshop and participated in its design and coordination. JW summarized the workshop discussion based on the invited lectures and the round-table discussions, JW and PY then converted this summary to a preliminary version of the manuscript. JW, PY and CA all participated in the revision process, and all read and approved the final manuscript.

## Pre-publication history

The pre-publication history for this paper can be accessed here:



## References

[B1] Paediatric HIV and treatment of children living with HIV. http://www.who.int/hiv/paediatric/en/index.html.

[B2] HIV Drug Resistance. http://www.who.int/hiv/drugresistance/en/.

[B3] Scaling up antiretroviral therapy in resource-limited settings: Treatment guidelines for a public health approach. http://www.who.int/3by5/publications/documents/arv_guidelines/en/.

[B4] HIV/AIDS Publications, Guidelines. http://www.who.int/hiv/pub/guidelines/en/index.html.

[B5] Early detection of HIV infection in infants andchildren. http://www.who.int/hiv/paediatric/EarlydiagnostictestingforHIVVer_Final_May07.pdf.

[B6] Scaling-up HIV testing and counselling (TC) services. http://www.who.int/hiv/topics/vct/toolkit/en/index.html.

[B7] Blower S, Gershengorn H, Grant R (2000). A tale of two futures: HIV and antiretroviral therapy in San Francisco. Science.

[B8] Blower S, Aschenbach A, Gershengorn H, Kahn J (2001). Predicting the unpredictable: transmission of drug resistant HIV. Nature Medicine.

[B9] Uncertainty and Sensitity Analysis. http://www.semel.ucla.edu/biomedicalmodeling/usa.asp.

[B10] Velasco-Hernandez J, Gershengorn H, Blower S (2002). Could widespread usage of combination antiretroviral therapy eradicate HIV epidemics?. The Lancet Infectious Diseases.

[B11] Blower S, Aschenbach A, Kahn J (2003). Predicting the transmission of drug-resistant HIV: comparing theory with data. The Lancet Infectious Diseases.

[B12] Blower S, Farmer P (2003). Predicting the public health impact of antiretrovirals: preventing HIV in developing countries. AIDScience.

[B13] Blower S, Ma L, Farmer P, Koenig S (2003). Predicting the impact of antiretrovirals in resource poor settings: preventing HIV infections whilst controlling drug resistance. Current Drug Targets – Infectious Disorders.

[B14] Blower S, Bodine E, Kahn J, McFarland W (2005). The impact of the ARV rollout on drug-resistant HIV in Africa: insights from empirical data & theoretical models. AIDS.

[B15] Vardavas R, Blower S (2007). The emergence of HIV transmitted resistance in Botswana: "when will the WHO detection threshold be exceeded?". PLoS ONE.

[B16] Wilson DP, Blower S (2006). Designing equitable antiretroviral allocation strategies in resource-constrained countries. PLoS Medicine.

[B17] Yan E, Zhang S, Jayaraman GC, Goedhuis NJ, Brooks JI, Merks H, Rekart ML, Wong E, Singh AE, Dawood M, Wood M, Laing E, Sandstrom P, Archibald CP (2007). Regional variations in the trends of transmitted HIV drug resistance in Canada. Public Health Agency of Canada Preprint.

[B18] Liu Y, Mullins JI, Mittler JE (2006). Waiting times for the appearance of Cytotoxic T-lymphocyte escape mutants in chronic HIV-1 infection. Virology.

[B19] Wang K, Mittler JE, Samudrala R (2006). Comment on "Evidence for positive epistasis in HIV-1". Science.

[B20] Smith RJ, Wahl LM (2005). Drug resistance in an immunological model of HIV-1 infection with impulsive drug effects. Bulletin of Mathematical Biology.

[B21] Smith RJ, Wahl LM (2004). Distinct effects of protease and reverse transcriptase inhibition in an immunological model of HIV-1 infection with impulsive drug effects. Bulletin of Mathematical Biology.

[B22] Heffernan JM, Wahl LM (2006). Natural variation in HIV infection: Monte Carlo estimates that include CD8 effector cells. Journal of Theoretical Biology.

[B23] Heffernan JM, Wahl LM (2006). Improving estimates of the basic reproductive ratio: Using both the mean and the dispersal of transition times. Theoretical Population Biology.

[B24] Heffernan JM, Wahl LM (2005). Monte Carlo estimates of natural variation in HIV infection. Journal of Theoretical Biology.

[B25] Alexander ME, Bowman CS, Feng Z, Gardam M, Moghadas SM, Röst G, Wu J, Yan P (2007). Emergence of drug-resistance: implications for antiviral control of pandemic influenza. Proc Biol Sci.

[B26] Newman MEJ (2003). The structure and function of complex networks. SIAM Rev.

[B27] Newman ME (2002). The spread of epidemic disease on networks. Phys Rev E Stat Nonlin Soft Matter Phys.

[B28] Watts DJ, Strogatz SH (1998). Collective dynamics of "small-world" networks. Nature.

[B29] Albert R, Barabasi AL (2002). Statistical mechanics of complex networks. Reviews of Modern Physics.

[B30] Glasser J, Taneri D, Thompson W, Chuang J, Wu J, Tull P, Alexander J (2007). Evaluation of targeted influenza vaccination strategies via population modeling. preprint.

